# Effect of Metronidazole on the Oxidoreductive Processes in the Submandibular and Parotid Glands in Experimental Research

**DOI:** 10.1155/2018/7083486

**Published:** 2018-10-24

**Authors:** Barbara Onopiuk, Paweł Onopiuk, Zofia Dąbrowska, Ewa Dąbrowska, Małgorzata Pietruska, Halina Car

**Affiliations:** ^1^Medical University of Białystok and Private Dental Office in Białystok, ul. Rzemieślnicza 37, Białystok 15-773, Poland; ^2^Otolaryngology Department, Medical University of Białystok, M. Skłodowskiej-Curie 24 A, 15-276 Białystok, Poland; ^3^Faculty of Medicine, Medical University of Białystok, M. Skłodowskiej-Curie 24 A, 15-276 Białystok, Poland; ^4^Department of Social and Preventive Dentistry of Medical University in Białystok, ul. Akademicka 3, Białystok 15-286, Poland; ^5^Department of Periodontology, Medical University of Białystok, ul. Waszyngtona 13, 15-269 Białystok, Poland; ^6^Department of Experimental Pharmacology, Medical University of Białystok, ul. Szpitalna 37, 15-295 Białystok, Poland

## Abstract

Oxidative stress takes part in the pathomechanisms of many diseases, including oral disorders. The imbalance between oxidative and antioxidative processes may lead to periodontitis, osteitis, or oral cancers. Furthermore, many chemotherapeutics, e.g., metronidazole (MTZ), may also cause toxic reactions and affect oxidative reactions. The research focused on MTZ influence on oxidative destruction in the parotid and submandibular gland tissue in animal experimental model. Therefore, the concentrations of enzymatic and nonenzymatic markers of oxidative stress were measured in these two rat glands in the control and experimental MTZ-treated groups. The material for analysis included parotid and submandibular glands of male Wistar rats, which were treated with metronidazole for 7 days by gastric tube in a dose of 100 mg/kg b.w. On day 8, the material was obtained and frozen in temp. −80°C. Then, the following seven enzymatic and nonenzymatic parameters were measured: GPx, TOS, TAS, SOD, LPO, CAT, and GSH. The data were analysed using Statistica 10.0. Metronidazole treatment in the experimental model showed an increase in LPO, TOS, and TOS/TAS and a decrease in CAT, SOD, GPx, and TAS. The conclusions of this research were made. Metronidazole treatment in a dose of 100 mg/kg b.w. caused imbalance between oxidative and antioxidative reactions in the rat parotid and submandibular glands. An increase was observed in LPO, TOS, and TOS/TAS in both glands exposed to metronidazole. Decreased activity of CAT, SOD, GPx, and TAS was noted, which indicates attenuation of the gland antioxidative protective barrier.

## 1. Introduction

Oxidative stress takes part in the pathomechanisms of many diseases, including oral disorders. The oxidoreductive status imbalance may promote periodontitis, osteitis, and oral cancers. Furthermore, mechanisms of toxic effects of many xenobiotics, including drugs, e.g., metronidazole (MTZ), may also be related to these disorders.

Metronidazole as a chemotherapeutic occurs in many forms and is applied locally (in the form of gel, ointment, or liquid) and systemically in the form of tablets or infusion fluids of various concentrations. It has been used primarily for the treatment of protozoan and anaerobic infections of various organs, including the oral cavity. The widespread use of MTZ in dentistry, in periodontal and endodontic treatment, and in dental surgery has drawn researchers' attention to adverse effects observed after its application in the oral cavity.

The increase in the amount of reactive oxygen species (ROS), caused by their excessive production or impairment of the body defence mechanisms, is referred to as oxidative stress. It leads to many disturbances in the physiology of cells and tissues at the molecular level, resulting in changes in the conformation and size of biological macromolecules [1].

Living organisms have developed defence mechanisms against toxic action of ROS, referred to as the antioxidant barrier. They consist of enzymatic and nonenzymatic antioxidants, which are also found in the saliva. Therefore, the saliva plays an important role in combating oxidative stress [2–4].

The total antioxidant potential of the saliva consists primarily of low-molecular antioxidants such as glutathione (GSH), ascorbic acid, uric acid, albumins, and the salivary peroxidase system, i.e., peroxidase secreted by the salivary glands and myeloperoxidase released from neutrophilic granules [2, 4–6].

Salivary peroxidase prevents the accumulation of hydrogen peroxide (H_2_O_2_) in the oral cavity and catalyses the oxidation of thiocyanates, bromides, and iodides. The anions and acids formed in this reaction (OSCN−, HOSCN) act to inhibit cells of bacteria, viruses, and yeasts. Myeloperoxidase catalyses the oxidation of chlorides, forming hypochlorous acid which is a strong germicidal substance [2]. However, the activities of superoxide dismutase (SOD), catalase (CAT), and glutathione peroxidase (GPx) in the saliva are minor compared to their activities in the blood [4, 6].

In recent years, researchers have focused on the involvement of oxidative stress in the pathomechanisms of many diseases, including those affecting the mouth and the masticatory system. Numerous studies have confirmed that disturbances in the oxidoreductive balance are causative factors of periodontal disease, bone disorders, and oral cancer [6, 7]. Moreover, the mechanisms of toxic actions of many xenobiotics, including such drugs as metronidazole, are associated with these disorders [8–12]. In experimental studies, the mutagenic potential of this drug has been demonstrated, which may be related to the reduction in the nitro group of MTZ and the formation of reactive oxygen species [13, 14].

Numerous studies have focused on the mutagenic potential of metronidazole, which according to their authors is related to the reduction in the nitro group and the emergence of reactive oxygen forms [8, 9, 13].

In the earlier studies, performed by authors of this paper, the similar research model was made on rats' salivary glands and the changes in oxidative stress potential. However, the research was concerned about the effect of increased zinc supply against oxidative damage of sublingual gland in chronic exposure to cadmium [12]. The amount of cadmium exposure at 5 mg and 50 mg Cd/dm3 induced oxidative stress in the sublingual glands of the rats. It reduced the TAS and GSH levels and increased LPO and TOS. In cadmium exposure conditions, increasing the supply of zinc by 79% or 151%, as compared to the standard dietary intake of this microelement, completely prevented the reduction of TAS and GSH levels and accumulation of LPO, H_2_O_2_, and TOS in the examined glands at both exposure levels to that metal. The outcome data revealed protective effect of increased zinc intake on the sublingual gland tissue in chronic cadmium exposure.

So far, metronidazole's impact on the development of stress in rat salivary glands has not been studied. Therefore, the decision was made to perform an experimental study to determine the possible effect of this drug on the development of oxidative damage in the submandibular and parotid glands in an experimental animal model. In our previous research, we were studying the impact of other xenobiotics on the salivary gland oxidative stress. However, the metronidazole impact on this topic was not studied before, although it is commonly used. Therefore, it seems to be interesting if metronidazole had any influence on salivary glands while being used as standard treatment.

In addition, numerous studies have noted the mutagenic potential of this drug, which according to the authors is related with the reduction of the metronidazole nitro group and the emergence of reactive oxygen species. So far, its influence on the development of stress in rat salivary glands has not been studied. In connection with the above, in this work, experimental studies were undertaken to determine the possible effect of this drug while being used as standard treatment on the development of oxidative damage in the submandibular and parotid glands in an experimental animal model.

For this purpose, the concentration of enzymatic and nonenzymatic markers of the oxidation-reduction state in both salivary glands of control rats and those receiving metronidazole was determined.

### 1.1. Aim

The aim of the research was to study the effect of metronidazole on the oxidoreductive processes in rats' submandibular and parotid glands. For this purpose, the concentrations of enzymatic and nonenzymatic markers of the oxidoreductive status were determined in the two salivary glands of rats in the control group and in the metronidazole group.

## 2. Materials and Method

The submandibular and parotid glands were collected and preserved as part of the experiment conducted at the Centre for Experimental Medicine (CEM) in Białystok. The experiments on animals and the use of the preserved material were approved by the Local Ethics Committee for Experiments on Animals in Bialystok (no. 12/2011 and 2/2014).

The experiment was carried out for 7 days on 14-month-old male Wistar (Crl: WI (Han)) rats with an initial body weight of approximately 490–530 g. Throughout the experiment, the animals were kept individually in cages, in experimental rooms in CEM, under standard breeding conditions (temperature 20+/−2°C, relative humidity 65%, 12-hour circadian cycle). The rats had unrestricted access to drinking water and pelleted breeding feed (Hersteller, Germany) containing 54% of carbohydrates, 36% of proteins, and 10% of fat. The animals were randomly divided into 2 groups of 8 individuals:

*(i) MTZ Group*. Rats received metronidazole intragastrically (tablets containing 250 mg of active substance, Polpharma) once a day at a dose of 100 mg/kg body weight in a volume of 0.5 mL of drinking water.

*(ii) Control Group*. Rats received drinking water intragastrically once a day in a volume of 0.5 mL in the same time regimes as in the MTZ group.

The MTZ dose was chosen based on literature data [9, 11, 15]. At the end of the experiment, 64 salivary glands, including 32 submandibular and 32 parotid (16 in each group), were collected from rats under anesthesia with ketamine (80 mg/kg) and xylazine (5 mg/kg). The biological material was frozen (−80°C) until analysis.

### 2.1. Evaluation of Enzymatic and Nonenzymatic Parameters in the Rat Salivary Glands

Glutathione peroxidase (GPx) activity was determined using the Bioxytech® GPx-340 TM Kit (OxisResearch™, Foster City, USA); catalases (CAT) were determined spectrophotometrically using the Aebi method [1]; superoxide dismutase (SOD) was analysed spectrophotometrically using the Superoxide Dismutase Assay Kit (Cayman Chemical Company, Ann Arbor, MI, USA). The concentration of glutathione (GSH) was determined spectrophotometrically using the Glutathione Assay Kit (Cayman Chemical Company, Ann Arbor, MI, USA). The concentration of lipid peroxidation (LPO) in salivary gland homogenates was measured using the Bioxytech® LPO-586TM Kit (OxisResearch™, Portland, USA).

The total antioxidative potential (TAS) of both salivary glands was assessed using the ImAnOx (TAS) Kit (Immundiagnostik AG, Bensheim, Germany). The total oxidative potential (TOS) was measured using the PerOx (TOS) Kit (Immundiagnostik AG, Bensheim, Germany).

### 2.2. Methods of Preparation of Salivary Gland Homogenates

In order to determine markers of the oxidoreductive status and total protein, 20% homogenates of submandibular and parotid glands were prepared.

The salivary glands (of known mass) were homogenized in cold 50 mM potassium phosphate buffer, pH 7.4. For each cm^3^ of the homogenate, 0.01 cm^3^ of 0.5 M butylhydroxytoluene (BHT) in acetonitrile was added to prevent tissue autoxidation. The homogenates were divided into 2 portions: one for SOD and GPx determination was centrifuged at 20,000 ×g for 30 minutes at 4°C; the other for CAT, GSH, LPO, TAS, and TOS was centrifuged at 700 ×g for 20 minutes at 4°C. The supernatant was then separated and stored frozen (−80°C).

### 2.3. Protein Concentrations in Salivary Gland Homogenates

The concentration of total protein was determined to express the concentration of the oxidoreductive status parameters as calculated per protein in salivary gland homogenates. The assays were performed with the BioMaxima Kit (Lublin, Poland) according to the manufacturer's instructions. The protein concentration was expressed in mg/mL.

### 2.4. Statistical Analysis

The statistical analysis of the results was carried out using the Statistica 10 software (StatSoft, Tulsa, USA). The results are presented as mean and standard error (SE) for 8 rats in each experimental group. In order to assess the statistical variability of differences between the experimental groups, a one-way analysis of variance (ANOVA) was carried out using Duncan's post hoc test. The comparison of the examined parameters between submandibular and parotid glands was performed using ANOVA with repeated measurements. Differences between the groups were considered statistically significant at *p* < 0.05.

## 3. Results

### 3.1. The Activity of Glutathione Peroxidase (GPx) in the Rat Submandibular and Parotid Glands

GPx activity in the submandibular and parotid glands in rats given metronidazole and in control animals is shown in Table 1. GPx activity in the submandibular and parotid glands in control rats was 28.120 ± 0.677 and 15.820 ± 0.722 mU/mg of protein, respectively, and was significantly higher in the submandibular than in the parotid gland.

Administration of metronidazole to animals caused a statistically significant decrease in the activity of this enzyme by 22% in the submandibular gland and by 14% in the parotid gland compared to the control group. In addition, in this group of rats, the enzyme activity was significantly lower in the parotid gland than in the submandibular gland.

### 3.2. Catalase Activity (CAT) in the Rat Submandibular and Parotid Glands

CAT activity in the submandibular and parotid glands of rats in the metronidazole and control groups is shown in Table 1. CAT activity in the submandibular and parotid glands of the control rats was 11.190 ± 0.410 and 16.100 ± 0.505 mU/mg of protein, respectively, and was higher in the parotid gland.

In animals treated with metronidazole, the activity of this enzyme was statistically significantly lower by 37% in the submandibular gland and by 41% in the parotid gland, as compared to the control group. Moreover, in this group of rats, the enzyme activity was significantly higher in the parotid gland than in the submandibular gland.

### 3.3. Superoxide Dismutase Activity (SOD) in the Rat Submandibular and Parotid Glands

SOD activity in the submandibular and parotid glands of metronidazole-treated and control animals is shown in Table 1. SOD activity in the submandibular and parotid glands of the control rats was 0.423 ± 0.019 and 0.438 ± 0.025 U/mg protein, respectively, and did not differ between the glands tested.

In the animals treated with metronidazole, the activity of this enzyme was statistically significantly lower by 27% in the submandibular gland and by 36% in the parotid gland as compared to the control group.

### 3.4. Concentration of Glutathione (GSH) in the Rat Submandibular and Parotid Glands

The concentration of GSH in the submandibular and parotid glands of metronidazole-treated and control animals is shown in Table 1. The GSH concentration in the submandibular and parotid glands of the control rats was 0.356 ± 0.019 and 0.406 ± 0.028 nmol/mg protein, respectively. Administration of metronidazole to animals did not affect the level of this low-molecular weight thiol in both salivary glands.

### 3.5. Concentration of Lipid Peroxides (LPO) in the Rat Submandibular and Parotid Glands

The levels of LPO in the submandibular and parotid glands in the metronidazole and control groups are presented in Table 1. The concentration of LPO in the submandibular and parotid glands of the control rats was 0.393 ± 0.025 and 0.341 ± 0.015 nmol/mg of protein, respectively, and did not differ between the salivary glands.

Administration of metronidazole to animals resulted in a statistically significant increase in LPO by 25% in the submandibular gland and by 51% in the parotid gland as compared to the control group.

### 3.6. Total Antioxidant Potential (TAS) in the Rat Submandibular and Parotid Gland

TAS concentration in the submandibular and parotid glands in metronidazole-treated and control animals is presented in Table 1. TAS concentration in the submandibular and parotid glands of the control rats was 13.190 ± 0.860 and 12.860 ± 1.087 nmol/mg of protein, respectively, and did not differ between the glands examined.

In the animals treated with metronidazole, TAS concentration, compared to the control group, was significantly lower by 63% in the submandibular gland and by 71% in the parotid gland.

### 3.7. Total Oxidative Potential (TOS) in the Rat Submandibular and Parotid Glands

TOS concentration in the submandibular and parotid glands in metronidazole-treated and control animals is shown in Table 1. TOS concentration in the submandibular and parotid glands in control rats was 57.650 ± 2.019 and 30.990 ± 2.419 nmol/mg protein, respectively, and was higher in the submandibular gland.

In the animals treated with metronidazole, the concentration of TOS was statistically significantly 1.5 times higher than in the control group in the submandibular gland and 2 times in the parotid gland.

### 3.8. Oxidative Stress Index (TOS/TAS) in the Rat Submandibular and Parotid Glands

The TOS/TAS index in the submandibular and parotid glands of metronidazole-treated rats and control animals is shown in Table 1. The TOS/TAS ratio in the submandibular and parotid glands of the control rats was 4.446 ± 0.194 and 2.487 ± 0.196, respectively.

In the animals treated with metronidazole, the TOS/TAS ratio was statistically significantly higher as compared to the control group by 4 times in the submandibular gland and 7 times in the parotid gland.

## 4. Discussion

Metronidazole as a chemotherapeutic occurs in many forms and is widely used in dentistry, for instance in periodontal treatment [16–19], endodontic treatment [20, 21], and in dental surgery [6, 22]. Data concerning the effect of MTZ on the oxidoreductive status of the rat salivary glands are not available in literature, thus hindering wider discussion of our results.

The antioxidant enzymes and low-molecular weight antioxidants showed varied activity in the saliva produced by the parotid glands and mucous saliva and mixed saliva secreted by the submandibular and sublingual glands [2, 6, 7].

The reported data seem to indicate that oxidative stress can be considered an important factor associated with oral diseases. Living organisms have developed defence mechanisms that are also present in the saliva. Due to its enzymatic and nonenzymatic defence systems, the saliva plays an important role in the fight against oxidative stress.

Researchers are also concerned with the side effects after MTZ application observed in various organs, including oral structures, such as the salivary glands. So far, the effect of MTZ on these glands has not been studied. In our research, MTZ administered to rats at a dose of 100 mg/kg of body weight induced oxidative stress in the tissues examined. The increase in lipid peroxidation (LPO), total oxidative potential (TOS), and stress index (TOS/TAS) in both salivary glands of rats receiving MTZ indicates excessive accumulation of ROS, whereas attenuated activities of catalase, superoxide dismutase, glutathione peroxidase, and total antioxidant potential (TAS) prove weak enzymatic antioxidant defence. These changes indicate a serious disturbance in the oxidoreductive balance in the salivary glands examined in the rats receiving MTZ.

It has been found that the level of salivary nonenzymatic antioxidants and antioxidant enzymes in the saliva of the parotid gland is higher in comparison with the saliva secreted by the submandibular and sublingual salivary glands [6]. Saliva from the parotid gland is the main source of antioxidants in the mouth. Higher antioxidant activity of this saliva is probably related to the presence of higher concentration of transition metals and its activity in the formation processes of food bite and digestion, while the secretion from the other two salivary glands (i.e., submandibular and sublingual) is constant and related to maintaining the integrity of the oral cavity. This, in turn, may suggest that the oral cavity is protected against RFT only partially and with varying force during the day. A varied composition of saliva produced by the salivary glands proves that their secretion plays a different role in different regions of the oral cavity [7].

In our current research, higher activities of CAT were found in the parotid gland; GPx activity was higher in the submandibular gland, while SOD, GSH, and LPO activities were the same in both glands examined.

The pharmacokinetics of MTZ in plasma has been elucidated and described, yet only few data are available on the penetration of the drug into the saliva and gingival fluid. Pähkla et al. [19] evaluated the concentration of MTZ in various body fluids after oral administration. The study was conducted in 11 patients with generalized periodontitis. MTZ was administered three times a day at a dose of 500 mg for two days. Serum, saliva, and gingival fluid samples were collected for further testing 2 hours after the last dose of the drug. The concentration of MTZ in biological fluids was determined by high-performance liquid chromatography. The mean drug concentrations in serum, saliva, and gingival fluid did not differ significantly (14.33, 15.15, and 12.86 ng/mL, respectively). The study showed that MTZ penetrates well into the saliva and gingival fluid. It can therefore be assumed that it also influenced the salivary glands evaluated in the current study.

During antimicrobial therapy, MTZ is often administered with other medicines, including aminoglycoside antibiotics, quinolones, rifampicin, or chloramphenicol, which can induce oxidative stress [23–25].

Kohanski et al. [24] in their research showed that aminoglycoside antibiotics generate the formation of hydroxyl radicals. In addition, literature reports indicate that ROS generated during the use of antibiotics play a key role in their bactericidal activity, although they also indicate a mutagenic effect not only in relation to bacterial cells [24, 25]. Vatansever et al. consider free radicals to be an attractive weapon against pathogens [25]. Strategies for using ROS in antimicrobial therapy are promising, but they should be further investigated, so that they can be used as an effective therapy in the future. On the other hand, Cornejo-Garcia et al. [23] investigated the oxidoreductive potential of the blood in patients with an allergic reaction to drugs, e.g., amoxicillin, cefaclor, and metamizole. The authors found that in the study group with symptoms of immune response, SOD activity was increased and GPx decreased. In addition, the concentrations of carbonyl proteins and lipid peroxidation were found to increase.

As mentioned earlier, the toxic effect of metronidazole is related to its metabolism in the human body to two major metabolites: 2-methyl-5-nitroimidazole-1 acetic acid (AAM) and 1-(2-hydroxyethyl)-2-hydroxy-5-nitroimidazole (HM). The HM metabolite has been shown to have a stronger genotoxic activity than the parent drug [13, 14, 26, 27]. This may be related to the nucleophilic substitution reaction of these metabolites, resulting from the disruption of the imidazole ring from DNA or with the reduction in the MTZ nitro group and the formation of ROS [13, 14, 27]. According to literature data, DNA damage occurs as a direct effect of ROS, which consequently leads to single or double DNA strand breaks and transformation of nitrogenous bases [1]. Taking into consideration the formation of ROS and DNA damage by MTZ, we decided to investigate the effect of this drug on the oxidoreductive status of the rat salivary glands.

As mentioned earlier, the data concerning the effect of MTZ on the oxidoreductive status of the rat salivary glands are not available in literature, thus hindering wider discussion of our results. The existing literature data analyse only oxidative damage to liver nuclei and intestines and changes in the level of enzymatic antioxidants in the serum of experimental animals [10, 15]. The results of the current study pertaining to stress induction by MTZ are confirmed by Kumari and Singh, Ligha and Paul, and Pang et al. [8–10].

In a study conducted by Kumari and Singh [8], 12-week-old male Swiss mice were administered MTZ orally at a dose of 500 mg/kg/day for 28 days. The authors also gave them the extract of terrestris (*Tribulus terrestris*) fruit (200 mg/kg/day), used to treat reproductive dysfunctions in males. The activity of SOD, CAT, GPx, and glutathione reductase (GR) and the concentration of malondialdehyde (MDA) as one of the final lipid oxidation products were determined in 10% of testicle homogenates.

In mice receiving metronidazole, the activity of SOD and MDA concentration significantly increased, while the activity of CAT, GPx, and GR decreased, which confirms the induction of stress by MTZ in this tissue. Moreover, the increased activity of SOD in combination with decreased activity of CAT and GPx leads to an increase in the level of H_2_O_2_ and its highly reactive derivative, i.e., hydroxyl radical. However, the increase in MDA concentration in the testicles of mice receiving MTZ suggests damage to the membranes of reproductive cells and their dysfunction. In addition, the administration of Tribulus fruit extract in mice was found to prevent MTZ from producing free radicals, and thus oxidative stress, as evidenced by the activity of antioxidant enzymes and LPO concentration returning to their control values. The protective effect of the extract is related to its ability to inhibit the formation of free radicals due to the presence of flavonoids, which have documented antioxidant properties.

In our study, both salivary glands showed lower activity of antioxidant enzymes after administration of MTZ as compared to the control rats. GPx, CAT, and SOD activities decreased, respectively, by 22% (*p* < 0.001), 27% (*p* < 0.05), and 33% (*p* < 0.001) in the submandibular gland and by 14% (*p* < 0.05), 36% (*p* < 0.001), and 41% (*p* < 0.001) in the parotid gland.

Some researchers point out the antioxidative action of MTZ. According to Rao et al. [11], burn wounds induce local oxidative stress, which lead to lipid peroxidation and further aggravate tissue destruction. These authors in their study conducted on male Wistar rats assessed whether metronidazole (180 mg/kg body weight) can reduce lipid peroxidation and promote healing of burn wounds. For this purpose, serum MDA was determined as one of the final products of lipid peroxidation, and the time of wound healing was specified in animals with burns and receiving MTZ. Metronidazole administration was shown to reduce MDA concentration threefold, which may indicate antioxidant effects of the drug. The results of the study by Rao et al. [11] may imply that the antioxidant effect of metronidazole increases its therapeutic efficacy.

The results of a study by Narayanan et al. [28] clearly suggest that metronidazole has antioxidant activity. The authors used cell-free lipid models of the skin. In the simple model, only linolenic acid was used as a dispersed aqueous solution, whereas in the combined model the liposomal system containing dipalmitoylphosphatidylcholine, cholesterol, and ceramide III was used in addition to linolenic acid. Solutions were prepared immediately before use. Lipid peroxidation was induced by UV radiation. The resultant MDA was quantified using the reaction with thiobarbituric acid. Various concentrations of metronidazole (10, 100, and 500 *μ*g/mL) were added to the control samples and after irradiation with UV rays. In the simple skin lipid model in the presence of 10, 100, and 500 *μ*g MTZ/mL, the concentration of MDA was reduced by 25, 36, and 49%, respectively. However, in the complex model in the presence of 100 and 500 mg MTZ/mL, the concentration of MDA was reduced by 19 and 34%, respectively. The results clearly indicate that metronidazole exhibits antioxidant properties in a cell-free environment.

Our results do not confirm the antioxidant effect of metronidazole. Metronidazole administered to rats at a dose of 100 mg/kg of body weight not only had no antioxidant effect but even induced stress in the salivary glands manifested as an increase in LPO concentration by 25% in the submandibular gland and 51% in the parotid gland. This may be due to the fact that the drug was administered to healthy animals and not as in the study conducted by Narayanan et al. in a closed system, deprived of cells, or in the study by Rao et al., where rats with burns were examined [11, 28].

Pélissier et al. [15] investigated the effect of metronidazole on the tissues of the small and large intestine and liver in inflammatory bowel disease (IBD). The cause of the disease may be not only antibiotic therapy but also oxidative stress. Therefore, the authors determined the concentration of oxidative damage markers of proteins (carbonyl proteins (PC)) and lipids (MDA) and glutathione as a nonenzymatic antioxidant in the intestinal tissues and liver of rats treated with metronidazole. They showed that treatment with this drug in a dose of 80 mg/kg b.w. for one week reduced statistically significantly PC concentration in the large intestine by 31% compared to the control group. In the colon, the level of GSH also decreased, which indicated antioxidant balance disorder. However, in the small intestine and liver, PC and GSH were not altered after administration of metronidazole. The authors suggest that GSH is present in most tissues in high concentrations, and diet is the main determinant of its homeostasis; hence, low levels of this antioxidant thiol were found in IBD patients but not in healthy people.

In our studies, both salivary glands showed no changes in the level of GSH, which, apart from uric acid, is the most important nonenzymatic antioxidant of the saliva classified as the first line of defence against ROS. The lack of changes in the level of GSH in the salivary glands is probably related to decreased GPx activity and to properly functioning thiol buffer ensuring a balance between the reduced and oxidized forms of this low-molecular weight thiol. Under physiological conditions, GSH is formed in hepatocytes from sulfuric amino acids (cysteine, methionine, and glutamate), which are provided with food. From the liver, it is secreted into the blood by hepatocytes. Its concentration in the saliva amounts to 2 *μ*M. Glutathione oxidizes its thiol group with free radicals, resulting in the formation of oxidized GSSG glutathione. Its activity is different in the saliva and in the fluid from the gingival crevices [2, 5].

Spolidorio et al. [29] studied the activity of SOD, GPx, and CAT and lipid peroxidation in the rat submandibular and parotid glands after the use of calcineurin inhibitors, such as cyclosporin (10 mg/kg mc) and tacrolimus (1 mg/kg mcg). Long-term, 60-day administration of immunosuppressive drugs resulted in a decrease in the activity of SOD, CAT, and GPx in both salivary glands and an increase in LPO, which indicated the induction of oxidative stress by these drugs. Similarly, a study by Kędzierska et al. [30] confirmed the influence of immunosuppressants on the development of oxidative stress in rats.

The available methods used to assess the oxidant and antioxidant balance also include the measurement of total oxidant potential (TOS) and total antioxidant potential (TAS) in various body tissues, including salivary glands [6, 31, 32]. TOS reflects the severity of stress in the body and the total amount of peroxides of all cellular macromolecules, while TAS is a parameter indicating the ability of antioxidants to remove free radicals and reduce their formation.

We showed a statistically significant decrease in TAS in the submandibular (*p* < 0.001) and parotid glands (*p* < 0.001) in rats receiving metronidazole, respectively, 2.8 and 3.5 times compared to control animals. In contrast, TOS increased 1.5 times (*p* < 0.001) in the submandibular gland and 2 times (*p* < 0.001) in the parotid gland. Also, the stress ratio presented as the TOS/TAS ratio was 4 and 7 times higher in animals receiving MTZ in the submandibular (*p* < 0.001) and parotid glands (*p* < 0.001), respectively. It should therefore be assumed that the local reduction in TAS is probably a consequence of a decrease in the activity of individual antioxidants during ROS inactivation. In the present study, the decreased activity of the enzymes tested was observed in both salivary glands in rats receiving MTZ.

In the light of the available literature data concerning damaging effects of metronidazole on salivary tissue, worthy of note is that the current study is the first to evaluate the effect of this drug on the oxidoreductive balance in the glands examined. The increase in LPO, TOS, and TOS/TAS index and a decrease in CAT, SOD, GPx, and TAS concentrations after MTZ administration demonstrated in our experimental model indicate the induction of oxidative stress by this drug in both salivary gland tissues.

### 4.1. Summary

The MTZ administered to rats at a dose of 100 mg/kg of b.w. disturbs the oxidoreductive balance in the submandibular and parotid glands. The increase in lipid peroxidation (LPO), total oxidative potential (TOS), and stress index (TOS/TAS) in both salivary glands of rats receiving MTZ indicates excessive accumulation of ROS. A reduction in the activity of catalase (CAT), superoxide dismutase (SOD), glutathione peroxidase (GPx), and total antioxidant potential (TAS) shows that the enzymatic antioxidant defence in both salivary glands is impaired.

### 4.2. Limitations and Future Research

The limitations during this project was the budget intended for the research. That conditioned the number of study groups and laboratory work. Furthermore, the main limitation of the study was limited publications in this subject that have similar animal model. Also, the research was executed on older rats. In addition, the study could be conducted only in an animal model. Therefore, this mechanism in the human body is not entirely clear and requires further research. In the future, more xenobiotics will be studied and its influence on oxidative stress.

## 5. Conclusions

MTZ occurs in many forms and is often used in dentistry in periodontal treatment, endodontic treatment, and in dental surgery. The results of our own research and studies conducted by other authors discussed in this paper have important practical implications, indicating the need for antioxidant actions during long-term therapy with this drug.

Summing up, the current study draws attention to the possibility of a damaging effect of MTZ in the rat submandibular and parotid glands.

## Figures and Tables

**Table 1:** Evaluation of enzymatic and nonenzymatic parameters in the rat salivary glands.

Parameters	Submandibular gland		Parotid gland	
	Control (C)	Metronidazole (MTZ)	Control (C)	Metronidazole (MTZ)
GPx (mU/mg protein)	28.120 ± 0.677	21.910 ± 0.942^∗∗∗^	15.820 ± 0.722^†††^	13.680 ± 0.595^∗^^†††^
CAT (mU/mg protein)	11.190 ± 0.441	7.478 ± 0.480^∗∗∗^	16.100 ± 0.505^†††^	9.436 ± 0.490^∗∗∗^^††^
SOD (U/mg protein)	0.423 ± 0.019	0.309 ± 0.042^∗^	0.438 ± 0.025	0.279 ± 0.016^∗∗∗^
GSH (nmol/mg protein)	0.356 ± 0.016	0.363 ± 0.010	0.406 ± 0.028	0.366 ± 0.016
LPO (nmol/mg protein)	0.393 ± 0.025	0.493 ± 0.025^∗^	0.341 ± 0.015	0.516 ± 0.063^∗∗∗^
TAS (nmol/mg protein)	13.190 ± 0.860	4.779 ± 0.423^∗∗∗^	12.860 ± 1.087	3.668 ± 0.303^∗∗∗^
TOS (nmol/mg protein)	57.650 ± 2.019	85.890 ± 3.822^∗∗∗^	30.990 ± 2.419^†††^	63.730 ± 2.748^∗∗∗^
TOS/TAS	4.446 ± 0.194	18.590 ± 1.092^∗∗∗^	2.487 ± 0.196	18.250 ± 1.645^∗∗∗^

^∗^Relative to the control group, ^∗^*p* < 0.05, ^∗∗∗^*p* < 0.001. ^†^Relative to the submandibular gland, ^†††^*p* < 0.001.

## Data Availability

The data used to support the findings of this study are available from the corresponding author upon request.
